# Whole-tumour evaluation with MRI and radiomics features to predict the efficacy of S-1 for adjuvant chemotherapy in postoperative pancreatic cancer patients: a pilot study

**DOI:** 10.1186/s12880-021-00605-4

**Published:** 2021-04-26

**Authors:** Liang Liang, Ying Ding, Yiyi Yu, Kai Liu, Shengxiang Rao, Yingqian Ge, Mengsu Zeng

**Affiliations:** 1 Department of Radiology, Zhongshan Hospital, Fudan University, and Shanghai Institute of Medical Imaging, No. 180 Fenglin Road, Xuhui District, Shanghai, 200032 China; 2 Department of Medical Oncology, Zhongshan Hospital, Fudan University, No. 180 Fenglin Road, Xuhui District, Shanghai, 200032 China; 3 Siemens Healthineers, No. 278 Zhou Zhu Road, Pudong New District, Shanghai, 201318 China

**Keywords:** Magnetic resonance imaging, Radiomics, Carcinoma, Pancreatic Ductal, Drug therapy, Survival analysis, Personalized medicine

## Abstract

**Background:**

Multiple guidelines for pancreatic ductal adenocarcinoma (PDAC) suggest that all stages of patients need to receive postoperative adjuvant chemotherapy. S-1 is a recently emerged oral antitumour agent recommended by the guidelines. However, which population would benefit from S-1 needs to be determined, and predictors of chemotherapy response are needed for personalized precision medicine. This pilot study aimed to initially identify whether whole-tumour evaluation with MRI and radiomics features could be used for predicting the efficacy of S-1 and to find potential predictors of the efficacy of S-1 as evidence to assist personalized precision treatment.

**Methods:**

Forty-six patients with PDAC (31 in the primary cohort and 15 in the validation cohort) who underwent curative resection and subsequently adjuvant chemotherapy with S-1 were included. Pre-operative abdominal contrast-enhanced MRI was performed, and radiomics features of the whole PDAC were extracted from the primary cohort. After univariable analysis and radiomics features selection, a multivariable Cox regression model for survival analysis was subsequently used to select statistically significant factors associated with postoperative disease-free survival (DFS). Predictive capacities of the factors were tested on the validation cohort by using Kaplan–Meier method.

**Results:**

Multivariable Cox regression analysis identified the probability of T_1_WI_NGTDM_Strength and tumour location as independent predictors of the efficacy of S-1 for adjuvant chemotherapy of PDAC (*p* = 0.005 and 0.013) in the primary cohort, with hazard ratios (HRs) of 0.289 and 0.293, respectively. Further survival analysis showed that patients in the low-T_1_WI_NGTDM_Strength group had shorter DFS (median = 5.1 m) than those in the high-T_1_WI_NGTDM_Strength group (median = 13.0 m) (*p* = 0.006), and patients with PDAC on the pancreatic head exhibited shorter DFS (median = 7.0 m) than patients with tumours in other locations (median = 20.0 m) (*p* = 0.016). In the validation cohort, the difference in DFS between patients with low-T_1_WI_NGTDM_Strength and high-T_1_WI_NGTDM_Strength and the difference between patients with PDAC on the pancreatic head and that in other locations were approved, with marginally significant (*p* = 0.073 and 0.050), respectively.

**Conclusions:**

Whole-tumour radiomics feature of T_1_WI_NGTDM_Strength and tumour location were potential predictors of the efficacy of S-1 and for the precision selection of S-1 as adjuvant chemotherapy regimen for PDAC.

## Background

Pancreatic cancer is one of the most important causes of death in cancer patients [1], and pancreatic ductal adenocarcinoma (PDAC) is the most common pathological type of pancreatic cancer. Due to high-grade malignancy, even if the tumour is detected early and curative resection is performed, as many as 60% of patients experience recurrence and metastasis within a short period of time after the operation [2, 3]. Multiple diagnostic and treatment guidelines suggest that all stages of PDAC patients need to undergo postoperative adjuvant chemotherapy [4–6].

The regimens commonly suggested for adjuvant chemotherapy in the guidelines for PDAC include gemcitabine, 5-fluorouracil (5-FU)/leucovorin, S-1, etc. [4–6]. S-1 is a newly developed oral antitumour agent consisting of tegafur (a prodrug of 5-FU), gimeracil [a potent dihydropyrimidine dehydrogenase (DPD) inhibitor], and oteracil (an inhibitor of the phosphorylation of 5-FU in the gastrointestinal tract). Tegafur is transformed into 5-FU in the liver after oral administration [7]. It has been reported that monotherapy with S-1 demonstrates noninferiority to commonly used gemcitabine in overall survival for locally advanced and metastatic pancreatic cancer [8]. For postoperative adjuvant chemotherapy, S-1 significantly extended both the overall and relapse-free survival of patients with resected pancreatic cancer compared with gemcitabine and might contribute to the improvement of patients’ quality of life with fewer adverse reactions [9].

How can a suitable postoperative regimen be personalized from a variety of chemotherapy regimens listed in the PDAC guidelines? Because adverse reactions are common and the proportion of patients benefiting from chemotherapy is not high [10, 11], it is urgent to determine how to identify patients who are more likely to benefit from certain adjuvant chemotherapy regimens and how to maintain their quality of life while pursuing better clinical outcomes. However, the guidelines have not provided answers to these questions or solutions to these problems. Currently, there is no definite standard for the selection of drugs in the adjuvant chemotherapy of PDAC [4], and predictors of chemotherapy response are needed for personalized precision medicine.

The same questions and problems exist in the clinical application of S-1, especially which population can benefit from postoperative adjuvant chemotherapy with S-1. Radiomics technology, which has emerged in recent years, offers important advantages for the assessment of tumour biology. Radiomics analysis can aid in evidence-based clinical decision making in oncologic management and help achieve individualized precision medical care [12–14]. In PDAC, most prior radiomics studies represented by texture analysis were based on CT imaging, which have been associated with survival in patients who underwent surgeries [15–17], but none of these studies mentioned or analysed the effects of treatments on survival after the surgeries. Only a few radiomics analysis studies on MRI have been performed in PDAC. Whether MRI and radiomics features could be used for the prediction of therapy response to adjuvant chemotherapy in postoperative pancreatic cancer patients has not been reported in previous literature.

This pilot study aimed to find potential predictors of the efficacy of S-1 by analysing the associations among the clinical data, MRI findings, and whole-tumour radiomics features of PDAC patients with distinct responses to S-1 in postoperative adjuvant chemotherapy and to provide initial evidence as basis of further studies to assist personalized precision treatment in postoperative patients with PDAC.

## Methods

### Patients

#### General clinical data

Our institutional ethics review board approved this retrospective study (No. B2018-266), and the requirement for written informed consent was conditionally waived. From January 2012 to September 2017, the diagnosis of PDAC was confirmed in 91 patients by surgery and pathological examination at our institute among the patients who underwent an abdominal contrast-enhanced MRI examination with the same scanner (Magnetom Aera, Siemens Healthcare, Germany, 1.5 T). After curative resection, 31 of them who subsequently received adjuvant chemotherapy with S-1 were included in this study as the primary cohort. The follow-up period was from the time of surgery to November 2018. The inclusion and exclusion criteria of our study were as follows. Within the same study and follow-up period, a total of 15 patients were enrolled using the same criteria that used for the primary cohort besides that the MRI examinations were performed on Magnetom Avanto (Siemens Healthcare, Germany, 1.5 T) as the independent validation cohort.

Inclusion criteria:

1. Patients received abdominal contrast-enhanced MRI examination with Magnetom Aera (Siemens Healthcare, Germany, 1.5 T) at our institute and were suspected of having pancreatic cancer; the image quality was satisfactory for the study;
2. Patients underwent curative resection of the tumour at our institute, and the diagnosis of PDAC was confirmed by pathological examination;
3. The pre-operative laboratory tests and operation were within 1 month from the date of the MRI examination;
4. Patients received postoperative adjuvant chemotherapy with S-1 and follow-up;
5. Clinical information including demographic characteristics, laboratory tests, surgery, chemotherapy regimen, pathological findings, and follow-up were collected.

Exclusion criteria:

1. Poor image quality that was not acceptable;
2. Patients underwent percutaneous transhepatic cholangiodrainage (PTCD), biliary stent placement or antitumour treatments before the MRI examination;
3. PDAC failed to be resected;
4. The pre-operative laboratory test or operation was over 1 month from the date of the MRI examination;
5. Clinical information was incomplete;
6. Adjuvant chemotherapy other than S-1, follow-up only, or unknown treatments;
7. Other reasons that patients should be excluded.

#### S-1 regimen

Chemotherapy with S-1 started within 8 weeks after the operation. The dosage was determined based on the body surface at 40–60 mg per dose with 2 doses per day. S-1 was orally administered after breakfast and dinner for 28 days, followed by 14 days of rest. This administration of S-1 was repeated every 6 weeks for up to four cycles until the disease progressed or until patients were intolerant.

#### Factor to evaluate the efficacy of S-1 for adjuvant chemotherapy of PDAC: disease-free survival (DFS) time

DFS was defined as the time from the date of surgery to that of the first recurrence of the disease, the date last known to have no evidence of disease, or the date of the most recent follow-up with no disease. Follow-up information was garnered from the medical and follow-up records in the electronic medical records (EMR) system and radiological information system (RIS) of our institute. Patients who had no recurrence at the last follow-up (November 2018) or who were lost to follow-up were treated as censored in the analyses.

Kaplan–Meier analysis was used to calculate the median DFS of the patients in our study. The efficacy of S-1 for adjuvant chemotherapy of PDAC was evaluated by the median DFS. The patients were divided into the non-response group and the response group by the median DFS.

### MRI protocol

All patients in the primary cohort underwent the abdominal contrast-enhanced MR examination on the same scanner (Magnetom Aera, Siemens Healthcare, Germany, 1.5 T). T_1_-weighted images with spoiled gradient-echo using volumetric interpolated breath-hold examination (VIBE) sequence, T_2_-weighted turbo spin-echo (TSE) sequence and DWI (b = 0, 500 s/mm^2^) using single-shot spin-echo echo-planar imaging were obtained before contrast was administrated. Dynamic contrast-enhanced MR images with T_1_-weighted images using VIBE sequences were obtained at the arterial phase (AP, 10 s after the trigger threshold reached), portal venous phase (PVP, 30 s after AP), and delayed phase (DP, 80–120 s after PVP) after injection of 0.1 mmol/kg Gadopentetate dimeglumine at a rate of 1–2 mL/s. Automatic tracking trigger scanning was applied for contrast enhancement, with the trigger points located in the abdominal aorta and the threshold set at a signal of 90. T_1_-weighted images using VIBE sequence performed on Magnetom Avanto (Siemens Healthcare, Germany, 1.5 T) was used for the validation. Detailed parameters of each sequence are shown in Table 1.

**Table 1:** Parameters of MRI sequences

Parameter	Primary cohort			Validation cohort
	T_1_WI (VIBE)	T_2_WI	DWI	T_1_WI (VIBE)
Repetition time (ms)	3.47	2800	5100	5.04
Echo time (ms)	1.36	95	55	2.31
Field of view (mm^2^)	308 × 380	308 × 380	297 × 380	308 × 360
Matrix	320 × 240	384 × 273	192 × 154	256 × 167
Section thickness (mm)	3	5.5	6	3.5
Fat suppression	Y	Y	Y	Y

### Extraction of whole-tumour radiomics features

#### Whole-tumour segmentation

**Fig. 1:**
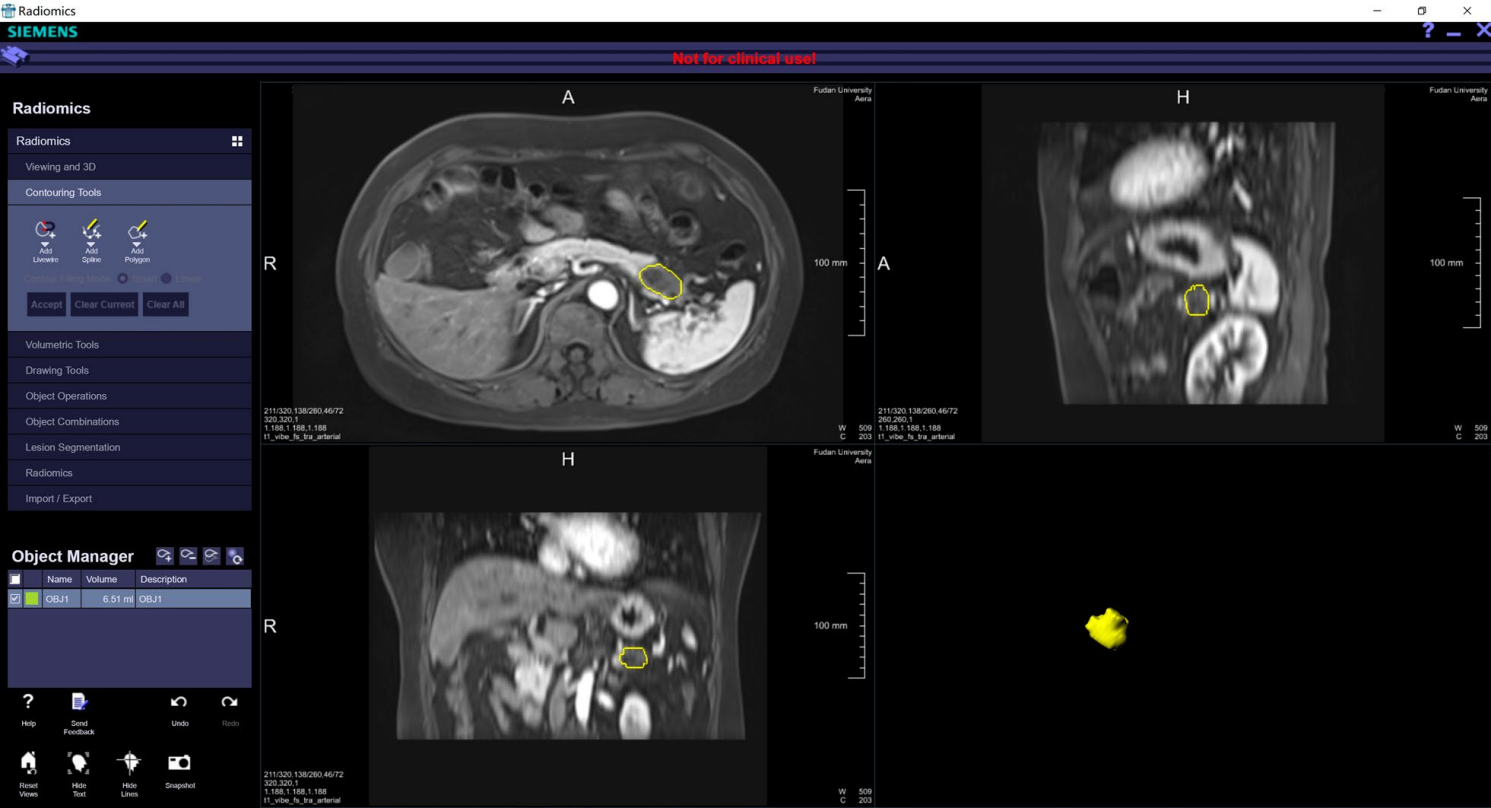
Whole-tumour radiomics analysis. Tumour segmentation was performed semi-automatically and whole-tumour radiomics features were extracted from the PDAC area (yellow overlay)

Whole-tumour segmentation was performed semi-automatically by a radiologist (with 11 years of experience in abdominal imaging), then checked and corrected by another radiologist (with 13 years of experience in abdominal imaging). A prototype software (Radiomics, Siemens Healthineers, Germany, not for commercial use) using a generic automatic segmentation algorithm based on a 3D domain in the workflow [18] was used for the segmentation. If the contour was not properly drawn, editing of the contour would be performed. A typical example of whole-tumour segmentation is presented in Fig. 1.

#### Extraction of radiomics features

All the segmentation data were subjected to radiomics feature extraction using the same prototype software interfacing with the Pyradiomics library [19]. A total of 110 radiomics features comprising 7 feature groups of whole-tumour were extracted from each studied sequence completely automatically: 19 first order statistics features, 16 contour-based features, 24 Grey Level Cooccurence Matrix (GLCM) features, 16 Grey Level Run Length Matrix (GLRLM) features, 16 Grey Level Size Zone Matrix (GLSZM) features, 5 Neighbouring Grey Tone Difference Matrix (NGTDM) features, 14 Grey Level Dependence Matrix (GLDM) features.

### Image analysis

The pre-operative PDAC imaging were evaluated subjectively and quantitatively. Signal of tumour, morphology of tumour, tumour margins, peripheral or central delayed enhancement of tumour, necrosis of tumour, peripancreatic infiltration, peripancreatic blood vessel invasion (arterial and venous), pancreatic duct dilatation, atrophy of the upstream pancreas and the presence of retention cyst [20] were evaluated by 2 radiologists (with 11 years and 13 years of experience in abdominal imaging) who were blinded to the clinical information on the picture archiving communication system (PACS). Consensus was achieved through discussion to resolve the controversy. Whole-tumour quantitative evaluation including the tumour size, ADC value of the whole tumour, enhancement rates of the whole tumour on AP, PVP and DP, as well as enhancement rate differences of the whole tumour between different dynamic contrast-enhanced phases [20] was performed by using data obtained from radiomics analysis.

### Statistical analysis

We performed the statistical analysis with SPSS statistical software (version 19.0.0, IBM, USA).

The demographic and clinical characteristics of the non-response group and the response group in the primary cohort were compared using Fisher's exact test (for categorical variables) or the Mann–Whitney *U* test (for continuous variables) to determine any relationship between the characteristics and the efficacy of S-1 for postoperative adjuvant chemotherapy of PDAC. The median DFS was calculated with the Kaplan–Meier method and compared by the Log Rank test. Univariable analysis with Fisher's exact test (for the subjective evaluation of pre-operative PDAC imaging, all of which were categorical variables) or the Mann–Whitney *U* test (for the whole-tumour quantitative evaluation of pre-operative PDAC imaging, all of which were continuous variables) was used for comparisons between the non-response group and the response group of the primary cohort to determine any association between pre-operative PDAC imaging findings and the efficacy of S-1 for postoperative adjuvant chemotherapy of PDAC.

Radiomics features selection: Univariable analysis with the Mann–Whitney *U* test method was adopted to select potentially valuable features to predict the efficacy of S-1 for postoperative adjuvant chemotherapy of PDAC. Features were selected from the whole-tumour radiomics features of T_1_WI (VIBE), T_2_WI, AP, PVP, and DP images from the primary cohort. The *p* value threshold was set at 0.05 for appropriate selection.

After univariable analysis and radiomics features selection, a multivariable Cox regression model (Cox proportional hazards model) of survival analysis was subsequently used to select statistically significant factors associated with postoperative DFS. Potentially significant variables with *p* value < 0. 05 in univariable analysis and selected radiomics features were enrolled in the analysis. A forward stepwise regression method based on maximum likelihood estimation (Forward: LR) was used for multivariable Cox regression analysis. Then, the patients were grouped by the factors selected through multivariable Cox regression analysis. Continuous variables were grouped by the cut-off value as the threshold, which was identified using receiver operating characteristic (ROC) curve analysis. Postoperative DFS between the subgroups was analysed using the Kaplan–Meier method and compared with the Log Rank test and survival curves to confirm the prediction performance of the selected factors. Predictive capacities of the selected factors were also tested on the validation cohort by using the Kaplan–Meier method and survival curves that evaluated by the Log Rank test.

*p* values < 0.05 were considered statistically significant and 0.05 ≤ *p* values < 0.1 were considered marginally significant in the study. In this pilot study we did not perform a correction for multiplicity given the small patient cohorts and exploratory nature of this study.

## Results

### Patient characteristics

The demographic information and clinical characteristics of the patients in the study are shown in Table 2. There were no differences found between the primary and validation cohorts, which enabled their use as primary and validation cohorts. The median DFS of the patients was 10.7 months in the primary cohort and 11.0 months in the validation cohort (*p* = 0.772). The patients in the primary cohort were divided into the non-response to S-1 group (DFS ≤ 10. 7 m, n = 16) and the response group (DFS > 10.7 m, n = 15) by the median DFS. The pre-operative CEA and CA19-9 levels in the non-response group were higher than those in the response group (*p* = 0.028 and 0.022). Tumour location was also different between the two groups (*p* = 0.032), i.e., the non-response group had a much higher proportion of tumours located on the head of the pancreas.

**Table 2:** Baseline demographic and clinical characteristics of patients

	Primary cohort (n = 31)			Validation cohort (n = 15)	*p* value (Inter cohorts)
	Non-response group (n = 16)	Response group (n = 15)	*p* value		
Age (years)^a^	64.25 ± 8.00	61.47 ± 8.63	0.259	63.40 ± 7.52	0.805
Sex			1.000		1.000
Male	8 (50.0%)	8 (53.3%)		7 (46.7%)	
Female	8 (50.0%)	7 (46.7%)		8 (53.3%)	
CEA (ng/mL)^b^	3.15 (2.55–5.73)	2.00 (1.60–2.80)	**0.028**	2.90 (2.30–4.70)	0.489
CA19-9 (U/mL)^b^	328.40 (120.78–469.38)	75.00 (45.80–166.30)	**0.022**	164.80 (22.70–429.50)	0.648
Tumour location			**0.032**		1.000
Pancreatic head	11 (68.8%)	4 (26.7%)		8 (53.3%)	
Pancreatic neck, body, and tail	5 (31.3%)	11 (73.3%)		7 (46.7%)	
Grade of tumour			1.000		1.000
2	5 (31.3%)	5 (33.3%)		5 (33.3%)	
3	11 (68.8%)	10 (66.7%)		10 (66.7%)	
Stage of tumour			0.585		0.848
I	6 (37.5%)	8 (53.3%)		6 (40.0%)	
II	7 (43.8%)	4 (26.7%)		5 (33.3%)	
III	3 (18.8%)	3 (20.0%)		4 (26.7%)	
Median DFS (months)	6.0	23.7	**< 0.001**	11.0	0.772

Bold means statistically significant (*p* values < 0.05)

^a^Mean ± standard deviation

^b^Median (P25–P75)

### Subjective evaluation of pre-operative MRI in the primary cohort

The findings of the subjective evaluation of pre-operative PDAC imaging are presented in Table 3. Two subjective factors of pre-operative MRI, irregular tumour morphology (43.8% vs. 6.7%) and peripancreatic tissue involvement (75.0% vs. 26.7%), were higher in the non-response group than in the response group of the primary cohort; the difference was statistically significant (*p* = 0.037 and 0.012). There seemed to be no differences in the other subjective evaluation variables of pre-operative MRI between the two groups in the primary cohort.

**Table 3:** Univariable analysis of subjective evaluation of pre-operative PDAC imaging findings and therapy efficacy of S-1 in the primary cohort

	Non-response group (n = 16)	Response group (n = 15)	*p* value
T_1_WI			1.000
Iso-intensity	1 (6.3%)	1 (6.7%)	
Mild hypo-intensity	15 (93.8%)	14 (93.3%)	
T_2_WI			1.000
Iso-/mild hypo-intensity	7 (43.8%)	7 (46.7%)	
Mild hyper-intensity	9 (56.3%)	8 (53.3%)	
Morphology			**0.037**
Regular	9 (56.3%)	14 (93.3%)	
Irregular	7 (43.8%)	1 (6.7%)	
Tumour margins on T_2_WI			0.394
Well defined	2 (12.5%)	4 (26.7%)	
Ill defined	14 (87.5%)	11 (73.3%)	
Tumour margins on T_1_WI			0.473
Well defined	5 (31.3%)	7 (46.7%)	
Ill defined	11 (68.6%)	8 (53.3%)	
Tumour margins after enhancement			0.073
Well defined	6 (37.5%)	11 (73.3%)	
Ill defined	10 (62.5%)	4 (26.7%)	
Peripheral delayed enhancement of tumour			0.135
Invisible	13 (81.3%)	8 (53.3%)	
Visible	3 (18.8%)	7 (46.7%)	
Central delayed enhancement of tumour			0.473
Invisible	8 (50.0%)	5 (33.3%)	
Visible	8 (50.0%)	10 (66.7%)	
Necrosis of tumour			0.172
Absence	11 (68.8%)	14 (93.3%)	
Presence	5 (31.3%)	1 (6.7%)	
Peripancreatic infiltration			**0.012**
Invisible	4 (25.0%)	11 (73.3%)	
Visible	12 (75.0%)	4 (26.7%)	
Peripancreatic blood vessel invasion			1.000
Invisible	10 (62.5%)	10 (66.7%)	
Visible	6 (37.5%)	5 (33.3%)	
Artery invasion			0.654
Invisible	14 (87.5%)	12 (80.0%)	
Visible	2 (12.5%)	3 (20.0%)	
Vein invasion			0.704
Invisible	10 (62.5%)	11 (73.3%)	
Visible	6 (37.5%)	4 (26.7%)	
Pancreatic duct dilatation			0.704
Absence	6 (37.5%)	4 (26.7%)	
Presence	10 (62.5%)	11 (73.3%)	
Atrophy of upstream pancreas			0.458
Absence	9 (56.3%)	11 (73.3%)	
Presence	7 (43.8%)	4 (26.7%)	
Retention cyst formation			–
Absence	16 (100.0%)	15 (100.0%)	
Presence	0 (0.0%)	0 (0.0%)	

Bold means statistically significant (*p* values < 0.05)

### Whole-tumour quantitative evaluation of pre-operative MRI in the primary cohort

The results of the whole-tumour quantitative evaluation of pre-operative PDAC imaging are presented in Table 4. Univariable analysis showed that two factors of whole-tumour quantitative evaluation, the enhancement rate of DP, the enhancement rate difference between DP and AP, were significantly different between the non-response group and the response group in the primary cohort (*p* = 0.013 and 0.036), both of which were lower in the non-response group. There seemed to be no differences in the other variables of the whole-tumour quantitative evaluation of pre-operative MRI between the two groups in the primary cohort.

**Table 4:** Univariable analysis of whole-tumour quantitative evaluation of pre-operative PDAC imaging and therapy efficacy of S-1 in the primary cohort

	Non-response group (n = 16)	Response group (n = 15)	*p *value
Short diameter (cm)	1.92 ± 0.71	1.84 ± 0.63	0.858
Long diameter (cm)	2.73 ± 1.17	3.16 ± 1.96	0.937
ADC value (10^–3^ mm^2^/s)	1.64 ± 0.53	1.85 ± 0.61	0.343
Enhancement rate of AP (%)	70.87 ± 26.78	88.98 ± 42.76	0.268
Enhancement rate of PVP (%)	141.08 ± 45.53	167.23 ± 50.48	0.114
Enhancement rate of DP (%)	158.29 ± 33.48	198.05 ± 45.97	**0.013**
Enhancement rate difference between PVP and AP (%)	70.22 ± 25.10	78.25 ± 31.96	0.607
Enhancement rate difference between DP and AP (%)	87.42 ± 26.08	109.07 ± 32.77	**0.036**
Enhancement rate difference between DP and PVP (%)	17.21 ± 29.44	30.82 ± 25.78	0.304

Bold means statistically significant (*p* values < 0.05)

### Whole-tumour radiomics features selection from the primary cohort

A total of 110 radiomics features of the whole tumour were extracted from each studied sequence of pre-operative MRI, and a total of 550 features were analysed for each PDAC lesion of the primary cohort. The results of whole-tumour radiomics features selection are shown in Table 5. Two features, including Complexity of NGTDM from T_1_WI (T_1_WI_NGTDM_Complexity) and Strength of NGTDM from T_1_WI (T_1_WI_NGTDM_Strength), were selected as potentially valuable features to predict the efficacy of S-1 for postoperative adjuvant chemotherapy of PDAC (*p* = 0.044 and 0.030). The mathematical formulas of the selected features are listed in the “Appendix”.

**Table 5:** Results of whole-tumour radiomics features selection from the primary cohort: based on therapy efficacy of S-1

Feature	Non-response group (n = 16)	Response group (n = 15)	*p *value
T_1_WI_NGTDM_Complexity^a^	0.00064 (0.00031–0.00116)	0.00131 (0.00065–0.00480)	**0.044**
T_1_WI_NGTDM_Strength^a^	0.021 (0.013–0.040)	0.038 (0.023–0.096)	**0.030**

Bold means statistically significant (*p* values < 0.05)

^a^Median (P25–P75)

### Survival analysis: factors relevant to therapy efficacy of S-1

After univariable analysis and radiomics features selection, CEA, CA19-9, tumour location, morphology of tumour, peripancreatic infiltration, enhancement rate of DP, enhancement rate difference between DP and AP, and the whole-tumour radiomics features of T_1_WI_NGTDM_Complexity and T_1_WI_NGTDM_Strength were enrolled into the multivariable Cox regression analysis. According to the results of the analyses, whole-tumour radiomics feature of T_1_WI_NGTDM_Strength and tumour location were significantly associated with postoperative DFS (*p* = 0.005 and 0.013), with hazard ratios (HRs) of 0.289 and 0.293, respectively (Table 6). Thus, these two factors may potentially predict the efficacy of S-1 for postoperative adjuvant chemotherapy of PDAC.

**Table 6:** Results of multivariable Cox regression analysis for factors relevant to therapy efficacy of S-1 in the primary cohort

	B	HR	95% CI	*p *value
T_1_WI_NGTDM_Strength	− 1.240	0.289	0.123–0.682	**0.005**
Tumour location	− 1.227	0.293	0.112–0.769	**0.013**

Bold means statistically significant (*p* values < 0.05)

Further survival analysis with the Kaplan–Meier method and the Log Rank test showed that grouping by T_1_WI_NGTDM_Strength or tumour location prompted significantly different postoperative DFS between the respective subgroups in the primary cohort (*p* = 0.006 and 0.016). The optimum cut-off value of T_1_WI_NGTDM_Strength generated by ROC curve analysis was 0.021 (*p* = 0.030, 95% confidence interval [CI], 0.551–0.907). Using this threshold value, the patients were classified into a low-T_1_WI_NGTDM_Strength group (value of T_1_WI_NGTDM_Strength < 0.021) and a high-T_1_WI_NGTDM_Strength group (value of T_1_WI_NGTDM_Strength ≥ 0.021).

**Fig. 2:**
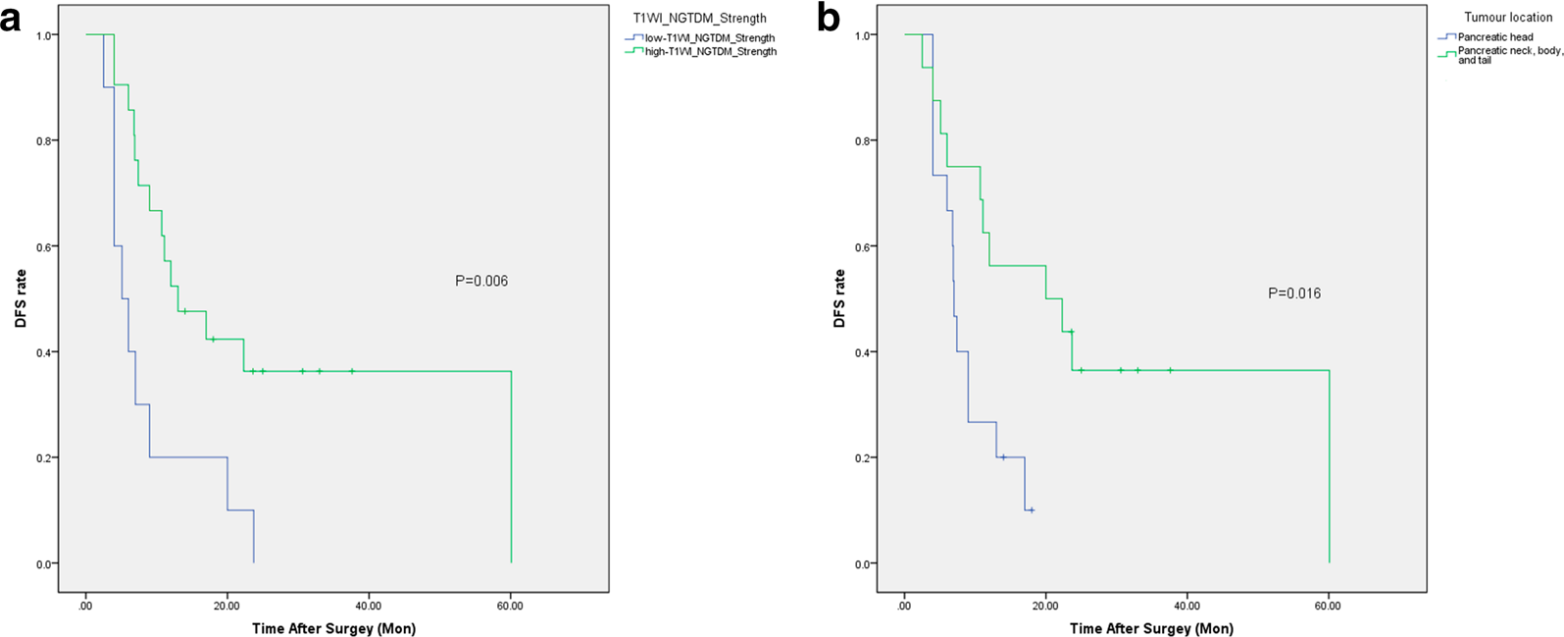
Survival analysis (DFS) for postoperative patients using adjuvant chemotherapy with S-1 in the primary cohort (K–M method). Grouping by T_1_WI_NGTDM_Strength (**a**) and tumour location (**b**) both prompted significantly different postoperative DFS between the respective subgroups

In the primary cohort, patients in the low-T_1_WI_NGTDM_Strength group had shorter DFS (median = 5.1 m) than those in the high-T_1_WI_NGTDM_Strength group (median = 13.0 m) (Table 7, Fig. 2a). Patients with PDAC on the pancreatic head exhibited shorter DFS (median = 7.0 m) than patients with PDAC in other tumour locations (median = 20.0 m) (Table 7, Fig. 2b). Whole-tumour radiomics feature of T_1_WI_NGTDM_Strength and tumour location showed significant value for predicting the efficacy of S-1 for postoperative adjuvant chemotherapy of PDAC independently.

**Table 7:** Results of survival analysis in the primary cohort: grouping by predictive factors

	Median DFS (months)	95% CI	*p* value
T_1_WI_NGTDM_Strength			**0.006**
Low-T_1_WI_NGTDM_Strength (n = 10)	5.1	2.0–8.2	
High-T_1_WI_NGTDM_Strength (n = 21)	13.0	4.5–21.5	
Tumour location			**0.016**
Pancreatic head (n = 15)	7.0	6.2–7.8	
Pancreatic neck, body, and tail (n = 16)	20.0	0.0–40.2	
Total (n = 31)	10.7	5.7–15.7	

Bold means statistically significant (*p* values < 0.05)

### Validation of the factors relevant to therapy efficacy of S-1

**Fig. 3:**
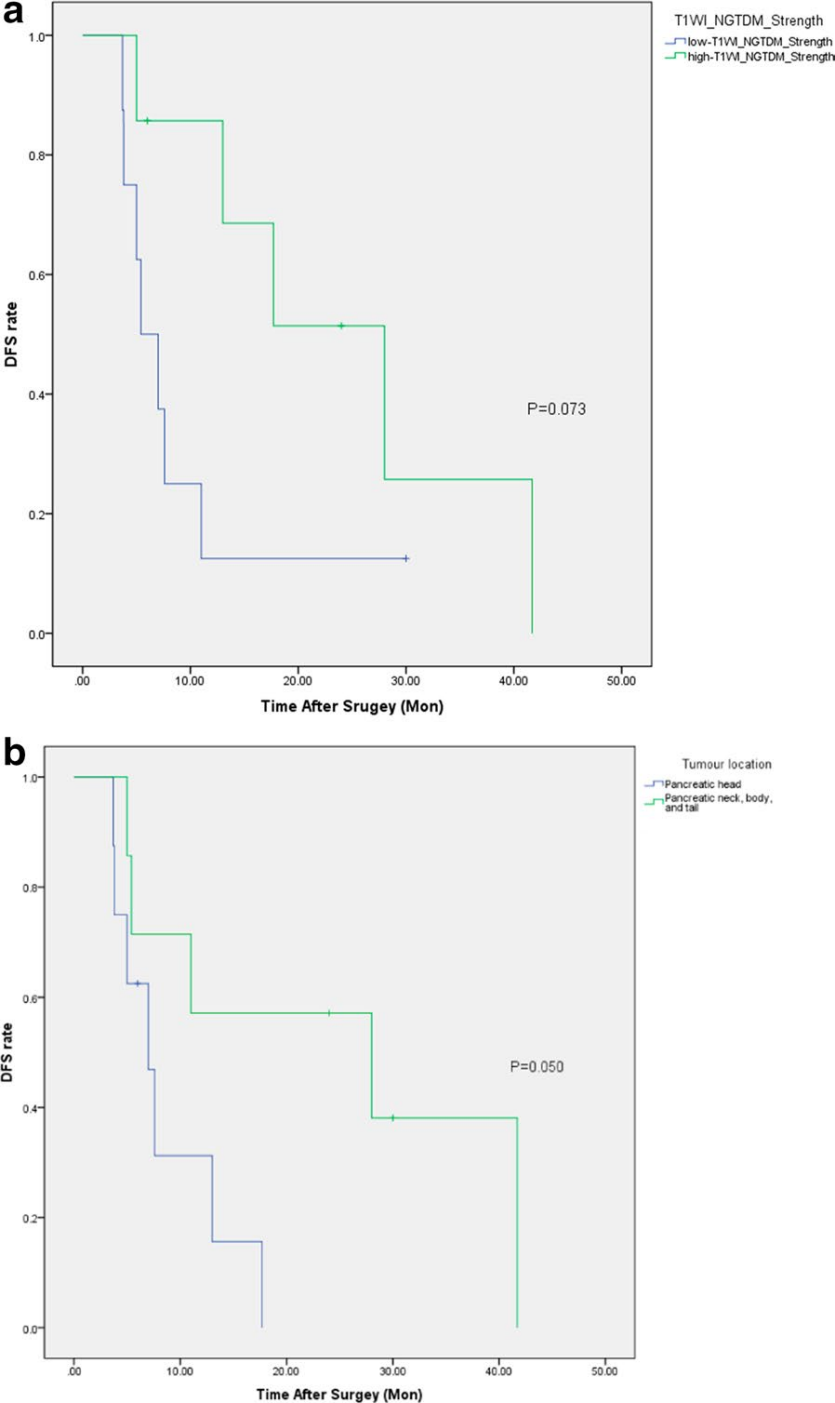
Survival analysis (DFS) for postoperative patients using adjuvant chemotherapy with S-1 in the validation cohort (K–M method). Grouping by T_1_WI_NGTDM_Strength (**a**) and tumour location (**b**) both prompted marginally different postoperative DFS between the respective subgroups

In the validation cohort, different subgroups of T_1_WI_NGTDM_Strength or tumour locations also prompted different postoperative DFS, with marginally significant (*p* = 0.073 and 0.050), respectively. Patients in the low-T_1_WI_NGTDM_Strength group had shorter DFS (median = 5.4 m) than those in the high-T_1_WI_NGTDM_Strength group (median = 28.0 m) (Table 8, Fig. 3a). Patients with PDAC on the pancreatic head exhibited shorter DFS (median = 7.0 m) than patients with PDAC in other tumour locations (median = 28.0 m) (Table 8, Fig. 3b).

**Table 8:** Results of survival analysis in the validation cohort: grouping by predictive factors

	Median DFS (months)	95% CI	*p* value
T_1_WI_NGTDM_Strength			0.073
Low-T_1_WI_NGTDM_Strength (n = 8)	5.4	2.6–8.2	
High-T_1_WI_NGTDM_Strength (n = 7)	28.0	13.7–42.3	
Tumour location			0.050
Pancreatic head (n = 8)	7.0	4.0–10.0	
Pancreatic neck, body, and tail (n = 7)	28.0	0.0–62.9	
Total (n = 15)	11.0	4.0–18.0

## Discussion

Multiple diagnostic and treatment guidelines suggest that all stages of PDAC patients need to undergo postoperative adjuvant chemotherapy [4–6]. As mentioned above, two important clinical studies in Asian populations confirmed the efficacy of S-1 for PDAC [8, 9], especially the superiority of S-1 to gemcitabine as postoperative adjuvant chemotherapy for PDAC in terms of overall survival. Additionally, compared to gemcitabine, S-1 has the convenience of oral administration, no complications of intravenous chemotherapy, good tolerability with fewer adverse reactions and might contribute to improving patients’ quality of life [9]. Currently, S-1 is recommended as a first-line adjuvant chemotherapy drug in the diagnostic and treatment guidelines for PDAC [5, 6].

However, the guidelines do not clearly indicate how to select the optimal chemotherapy regimen for PDAC patients suitably and individualizedly, and which population can benefit from S-1 is still unknown; proper evidence-based guidance is still lacking. Some studies proposed that the intratumoural expression levels of DPD and thymidylate synthase (TS) might be related to therapeutic outcomes in PDAC patients receiving S-1 chemotherapy [21–23], but the conclusion remains controversial; some studies even had contradictory results [24, 25].

In our study, the non-response to S-1 group had a much higher proportion of PDAC located on the pancreatic head than the response group in the primary cohort. In the validation cohort, patients with PDAC on the pancreatic head also exhibited shorter DFS. Previous studies also indicated that pancreatic head cancers and pancreatic body/tail cancers had different overall survival rates and tumour-free survival [26, 27]. Their biological characteristics, such as the concentration of cell-of-origin and microRNA expression, were also different [27]. These differences may be related to the embryonic development of the pancreas. The pancreas develops from the formation of a ventral bud and a dorsal bud, each with its own duct originating from the primitive intestine, that finally fuse as the pancreas. Thus, the blood supply, lymphatic backflow, and innervations between the head and body/tail of the pancreas are significantly different [28]. This may be the reason why the biological behaviours and the treatment responses of PDAC in different locations also differ.

To date, there are limited data on the effect of tumour location on the benefit from adjuvant chemotherapy, as most studies have not yet analysed the different responses to chemotherapy of tumours in different locations [28, 29]. Our study has partially filled that research gap, even though the sample size was relatively small. In our study, S-1 used for postoperative adjuvant chemotherapy of PDAC was much more effective for patients with tumours located in areas other than the pancreatic head, suggesting that the selection of S-1 for PDAC located on the pancreatic head should be made more cautiously. Interestingly, another study found that gemcitabine improved overall survival in a subgroup of postoperative patients with pancreatic head tumours compared to 5-FU [30, 31].

Because of the strict requirements for the consistency of treatment regimen and follow-ups in the enrolment criteria, the number of patients included in this pilot study was relatively small. Consequently, we only performed radiomics features screening and selection; multi-factor fusion model construction, which could be carried out in radiomics analysis, was not performed in our study. However, the identified radiomics feature-T_1_WI_NGTDM_Strength-was confirmed by a multivariable Cox proportional hazards model and Kaplan–Meier analysis to be a potential predictor for the efficacy of S-1 for postoperative adjuvant chemotherapy of PDAC. NGTDM_Strength is one of the radiomics features from the group of Neighbouring Grey Tone Difference Matrix (NGTDM). As high-order statistical parameters, NGTDM examines the signal intensity and spatial interrelationship between neighbouring voxels between adjacent image planes, which describe the dynamic range of intensities at a local level and better quantify the heterogeneity within the tumour [32]. Strength is a measure of the primitives in the image. Radiomics features can uncover tumour characteristics that may fail to be appreciated by the naked eye [33]. From the results of our study, NGTDM_Strength from T_1_WI was independently related to the therapy response of PDAC, supporting that radiomics features could potentially reflect the biological information of tumours, including the intratumoural heterogeneity associated with the response to treatment and survival [14, 34, 35]. In another diffusion-weighted-MRI-derived radiomics study, 13 radiomics features were found to be important for predicting the gemcitabine-based chemotherapy response of PDAC [36]. NGTDM_Strength was not included, demonstrating the possible specificity of NGTDM_Strength for predicting the efficacy of S-1, but the relationship between the radiomics features and therapy response to S-1 still needs to be further analysed in larger cohorts.

A few studies have assessed the potential of imaging data, including radiomics features, for the prediction of survival in PDAC with different examination techniques. One study showed that not only for resectable PDAC but also for locally advanced and/or metastatic PDAC, specific CT radiomics feature was a significant prognostic factor [37]. Another study that first evaluated the prognostic value of FDG-PET radiomics in pancreatic cancer found that feature of GLZLM GLNU was the most relevant factor for predicting 1-year survival [38]. However, these studies did not analyse the treatment response of specific regimens. Compared with most previous texture analysis studies in PDAC that were based on CT imaging [15–17, 39, 40], we performed radiomics analysis based on MRI, which has a higher resolution in soft tissue than CT. Although based on different examination techniques, the selected features are mostly related to intratumoural heterogeneity [32, 37, 38]. However, performances have not yet been compared among CT, MRI, and PET/CT [41, 42]. It is difficult to determine which examination technique is better for radiomics analysis from previous studies. MRI-based radiomics analysis may likely be more predictive of tumour heterogeneity but might be more susceptible to variations in imaging parameters compared to CT [41]. We performed all the MRI examinations of the same cohort with the same scanner in our study to avoid the influences of equipment and parameter differences.

Additionally, the univariable analysis demonstrated that the non-response to S-1 group had more cases of irregular tumour morphology and peripancreatic tissue involvement on pre-operative MRI, which reflected the higher invasiveness of PDAC in this group than in the response group. Although the multivariable Cox proportional hazards model did not show an association of these imaging signs and postoperative DFS with adjuvant chemotherapy of S-1, if these signs are present in pre-operative image assessment, the selection of S-1 warrants great caution, and a more intensive adjuvant chemotherapy regimen may be more appropriate. The above recommendation still needs to be further validated by studying larger cohorts.

The region of interest (ROI) of the whole tumour was conducted during the analysis in this study. Compared with the largest cross-sectional area analysis that was performed in several previous studies [17, 39], whole-tumour analysis is more representative of intratumoural biological characteristics and could avoid selection bias. The results are also relatively more reliable [43]. Therefore, whole-tumour imaging analysis including radiomics features may obtain additional predictive factors about the outcomes of treatments noninvasively and no extra examinations and medical expenses will be added. To the best of our knowledge, there are no similar studies about the imaging evaluation of PDAC in the literature. The results of our study could be the feasible basis of evidence and further studies for personalized precision medicine to select the right treatment for the right patient at the right time, thereby reducing the adverse reactions of ineffective treatments and improving the quality of life as well as prognosis of PDAC patients.

Our study has several limitations. Due to the strict requirements for the consistency of treatment regimen and follow-ups, the number of enrolled patients was relatively small and our study was a pilot study. In addition, the follow-up period was relatively short. Despite these limitations, this pilot study found that whole-tumour radiomics feature of T_1_WI_NGTDM_Strength and tumour location had significant value in predicting the efficacy of S-1 for postoperative adjuvant chemotherapy of PDAC independently in the primary cohort. However, the differences of DFS between different subgroups in the validation cohort were marginally significant with the same survival trends as the primary cohort. The reason for this might be that the sample size was not large enough to make the selected factors achieve steady performance. Another cause could be the different MRI scanners used in image acquisition in two cohorts, since radiomics features are known to vary with scanner type. Prospective research with a large sample size and different institutes is needed to further verify the reliability and repeatability of the predictors. Further studies to identify predictors for the selection of S-1 as chemotherapy regimen for locally advanced and metastatic pancreatic cancer are also needed.

## Conclusions

Our study has demonstrated that whole-tumour evaluation with MRI and radiomics features could be used for predicting the efficacy of S-1, along with that the whole-tumour radiomics feature of T_1_WI_NGTDM_Strength and tumour location were potential predictors of the efficacy of S-1 and for the precision selection of S-1 as adjuvant chemotherapy regimen of PDAC.

## Data Availability

The datasets used and/or analysed during the current study are available from the corresponding author on reasonable request.

## Appendix

The mathematical formulas of the selected features in part 4 of the results are as follows:

$${\text{Complexity of NGTDM}} = \frac{1}{{N_{v,p} }}\sum\limits_{i = 1}^{Ng} {\sum\limits_{i = 1}^{Ng} {\left| {i - j} \right|} } \frac{{p_{i} s_{i} + p_{j} s_{j} }}{{p_{i} + p_{j} }},$$

where *p*_*i*_ ≠ 0, *p*_*j*_ ≠ 0

$${\text{Strength of NGTDM}} = \frac{{\sum\nolimits_{i = 1}^{{N_{g} }} {\sum\nolimits_{j = 1}^{{N_{g} }} {(p_{i} + p_{j} )(i - j)^{2} } } }}{{\sum\nolimits_{i = 1}^{{N_{g} }} {s_{i} } }},$$

where *p*_*i*_ ≠ 0, *p*_*j*_ ≠ 0 (Only the mathematical formulas of the selected features are listed. For the mathematical formulas of the remaining studied radiomics features, see https://pyradiomics.readthedocs.io/en/latest/features.html).

## Abbreviations

PDAC: Pancreatic ductal adenocarcinoma
DFS: Disease-free survival
HR: Hazard ratio
DPD: Dihydropyrimidine dehydrogenase
PTCD: Percutaneous transhepatic cholangiodrainage
EMR: Electronic medical record
RIS: Radiological information system
TSE: Turbo spin-echo
VIBE: Volumetric interpolated breath-hold examination
ADC: Apparent diffusion coefficient
GLCM: Grey Level Cooccurence Matrix
GLRLM: Grey Level Run Length Matrix
GLSZM: Grey Level Size Zone Matrix
NGTDM: Neighbouring Grey Tone Difference Matrix
GLDM: Grey Level Dependence Matrix
PACS: Picture archiving communication system
AP: Arterial phase
PVP: Portal venous phase
DP: Delayed phase
ROC: Receiver-operating characteristic
TS: Thymidylate synthase
ROI: Region of interest
